# Null mutation of chloride channel 7 (*Clcn*7) impairs dental root formation but does not affect enamel mineralization

**DOI:** 10.1007/s00441-015-2263-z

**Published:** 2015-09-08

**Authors:** Jing Guo, Theodore J. M. Bervoets, Kim Henriksen, Vincent Everts, Antonius L. J. J. Bronckers

**Affiliations:** Department Oral Cell Biology, Academic Center of Dentistry Amsterdam (ACTA), University of Amsterdam and VU-University of Amsterdam, MOVE Research Institute, Gustav Mahlerlaan 3004, 1081 LA Amsterdam, The Netherlands; School of Stomatology/Dental Clinic, Zhejiang Chinese Medical University, Mailbox 97, Binwen Road 548, Binjiang District 310053 Hangzhou, China; Nordic Bioscience Biomarkers and Research A/S, Hovedgade 207, 2730 Herlev, Denmark

**Keywords:** ClC-7, Ameloblasts, Tooth eruption, Micro-CT, Immunostaining, Histomorphometry

## Abstract

ClC-7, located in late endosomes and lysosomes, is critical for the function of osteoclasts. Secretion of Cl^−^ by the ruffled border of osteoclasts enables H^+^ secretion by v-H^+^-ATPases to dissolve bone mineral. Mice lacking ClC-7 show altered lysosomal function that leads to severe lysosomal storage. Maturation ameloblasts are epithelial cells with a ruffled border that secrete Cl^−^ as well as endocytose and digest large quantities of enamel matrix proteins during formation of dental enamel. We tested the hypothesis that ClC-7 in maturation ameloblasts is required for intracellular digestion of matrix fragments to complete enamel mineralization. Craniofacial bones and developing teeth in *Clcn7*^-/-^ mice were examined by micro-CT, immunohistochemistry, quantified histomorphometry and electron microscopy. Osteoclasts and ameloblasts in wild-type mice stained intensely with anti-ClC-7 antibody but not in *Clcn7*^-/-^ mice. Craniofacial bones in *Clcn7*^-/-^ mice were severely osteopetrotic and contained 1.4- to 1.6-fold more bone volume, which was less mineralized than the wild-type littermates. In *Clcn7*^-/-^ mice maturation ameloblasts and osteoclasts highly expressed Ae2 as in wild-type mice. However, teeth failed to erupt, incisors were much shorter and roots were disfigured. Molars formed a normal dental crown. In compacted teeth, dentin was slightly less mineralized, enamel did not retain a matrix and mineralized fairly normal. We concluded that ClC-7 is essential for osteoclasts to resorb craniofacial bones to enable tooth eruption and root development. Disruption of *Clcn7* reduces bone and dentin mineral density but does not affect enamel mineralization.

## Introduction

Maturation ameloblasts, epithelial cells that deposit and mineralize dental enamel, have some structural and functional similarities with bone-resorbing osteoclasts. Osteoclasts and ameloblasts both have a ruffled border facing a calcified extracellular matrix and both secrete enzymes to degrade and remove this matrix (Josephsen and Fejerskov 1977; Salama et al. 1989, 1990; Zhao and Patrick Ross 2007). Both also have a similar pH-regulatory machinery including carbonic anhydrase-2, anion exchanger-2 (Ae2), v-H^+^-ATPase (Josephsen et al. 2010; Lin et al. 1994) and sodium bicarbonate cotransporter-1(Nbce1) (Jalali et al. 2014; Josephsen et al. 2010). Osteoclasts use this pH regulatory machinery to produce and secrete protons at their ruffled border with a central role for v-H^+^-ATPase. Maturation ameloblasts that mineralize dental enamel also express v-H^+^-ATPase and it has been proposed that these cells secrete protons to prevent precocious mineralization at the enamel surface that would otherwise inhibit deeper layers of enamel to mineralize (Josephsen et al. 2010). However, mineralization of enamel in mice with null mutation of *Tcirg1* (the osteoclast-specific subunit of the v-H^+^-ATPase proton pump in the ruffled border), was not different from wild-type enamel, suggesting that the v-H^+^-ATPase detected in maturation ameloblasts was another type of v-H^+^-ATPase involved in acidification of intracellular vesicles and trafficking rather than a plasma membrane-associated proton pump to secrete protons as in osteoclasts (Bronckers et al. 2012).

ClC-7 belongs to the CLC family of chloride channels and transporters, which consists in nine mammalian members with diverse physiological roles (Stauber et al. 2012). The CLC family comprises both plasma membrane-localized chloride channels and chloride-proton exchangers that reside predominantly in membranes of compartments of the endocytic pathway (Jentsch 2008; Stauber et al. 2012). ClC-7, a Cl^−^/ H^+^ antiporter (Leisle et al. 2011) and its β-subunit Ostm1 localize to lysosomes of all cells and additionally reside at the ruffled border membrane of osteoclasts (Kornak et al. 2001; Lange et al. 2006). ClC-7 in parallel to the v-type H^+^-ATPase is important for the acidification of the resorption lacuna (Kornak et al. 2001). In contrast to its effect on the pH in the resorption lacuna, the lysosomal pH is *not* changed in cells lacking ClC-7 (Kasper et al. 2005; Steinberg et al. 2010); lysosomal acidification is perhaps enabled by cation efflux (Steinberg et al. 2010). ClC-7 seems rather involved in Cl^−^ accumulation in lysosomal vesicles (Weinert et al. 2010).

Disruption of the *Clcn7* gene in mice results in severe osteopetrosis in the long bones of the extremities, shorter stature and splenomegaly (Kornak et al. 2001). Failure of teeth to erupt has also been reported for *Clcn7*^-/-^ mice but the description was not very detailed (Kasper et al. 2005; Kornak et al. 2001; Wen et al. 2015).

Ameloblasts secrete proteases as MMP20 and KLK4 to degrade most of the enamel matrix followed by endocytosis and digestion of at least a portion of the peptide fragments, particularly during maturation stage. Retention of the enamel matrix and incomplete enamel mineralization happen in mice with null mutation of *Mmp20*, *Klk4* or *Sppl2a* (an intramembranous protease located in lysosomes) (Bartlett et al. 2004; Bronckers et al. 2013; Simmer et al. 2009). Transcripts for nine CLC members have been identified in mouse ameloblasts, in the maturation stage most abundantly *Clcn*7 and ClC-7 protein was immunolocated in ameloblast vesicles (Lacruz et al. 2013). It was unknown whether disruption of *Clcn7* affects formation of enamel and dentin or changes the expression of other important chloride exchangers.

In view of the heterogeneity of osteoclasts, it was also unknown whether in *Clcn7*^-/-^ mice *all* bony structures are affected. Osteoclasts in the craniofacial bones differ in some aspects from those in long bones (Everts et al. 2009). In pycnodysostosis, the *Ctsk* mutation leads to sclerosis of the long bones but not in craniofacial bones (Gowen et al. 1999; Saftig et al. 2000). Null mutation of *Ae2a*,*b* causes osteopetrosis in long bones, not in craniofacial bones and disrupts formation of dental enamel (Jansen et al. 2009).

In this study, we address two questions: (1) Is a functional ClC-7 required for normal formation of teeth, in particular for different stages of amelogenesis and root formation? (2) Does disruption of *Clcn*7 also affect the function of osteoclasts in craniofacial bones resulting in osteopetrosis? We examine the functional relevance of ClC-7 for the development of teeth and craniofacial bones by studying changes in the structure of dental and bone tissues in *Clcn7*^-/-^ mice (Kornak et al. 2001; Neutzsky-Wulff et al. 2008).

## Materials and methods

### Mice

Heads and bones were obtained from six mice of the genetically modified *Clcn7* mouse strain and six of their wild-type littermates (courtesy Dr. T.J.Jentsch, Berlin, Germany). The *Clcn7* null mouse strain was generated by deletion of exons 3–7 in the *Clcn7* gene, which completely abolished expression of ClC-7 (construct C7A) as described by Kornak et al. (2001). Pups were fed with a soft (gel) diet each day after weaning until sacrificed at ages post-natal days 22 or 23. Three pairs of the mice were used for ultrastructural studies and another three pairs were used for micro-CT analysis, immunolocalization and histomorphometric analysis. All procedures were approved by the Committee for Animal Health and Animal Care of the VU-University and FMP/MDC in Berlin, Germany, according to national standards.

### Tissue fixation, embedding and sectioning

Mice were perfused first with phosphate-buffered saline (PBS) and then with 4 % formaldehyde in PBS. Heads and bones were collected and postfixed in 4 % formaldehyde in PBS overnight at 4 °C. From each group of 3 mice, mineralized tissues were scanned for micro-CT analysis. The samples were then decalcified in 4.18 % EDTA + 0.8 % formalin at pH 7.2 for 4 weeks at 4 °C, rinsed with phosphate buffer, embedded in paraffin and serially sectioned into 6-μm-thick sections for immunostaining and staining with hematoxylin-eosin (HE). For ultrastructural studies, mouse heads of *Clcn7*^-/-^ mice and wild-type controls were fixed in 1 % glutaraldehyde and 4 % formaldehyde in 0.1 M sodium cacodylate buffer pH 7.3 for 1–2 weeks. Then, the tissues were demineralized in 4.2 % EDTA + 0.8 % formaline for 2 weeks. After postfixation in 1 % OsO_4_ for 1 h, the tissues were dehydrated in ethanol and embedded in epoxy resin (LX112).

### Immunohistochemistry

EDTA-decalcified, paraffin-embedded tissue sections were immunostained with affinity purified rabbit anti-human ClC-7 (Abcam, catalogue ab31264) and rabbit anti-Ae2 (courtesy Dr. S. Kellokumpu, University of Oulu, Finland). According to the manufacturer, anti-ClC-7 antibody was raised against a synthetic peptide-conjugate to KLH from within amino acid residues 750 to C-terminus of the human ClC-7, with a predicted 94 % identity with mouse, rat and rabbit. Sections were deparaffinized in xylene, rehydrated in ethanol and washed in Tris-buffered saline (TBS). Antigen retrieval was carried out for both antibodies by incubation in 10 mM citrate buffer (pH 6.0) overnight at 60 °C. Nonspecific staining of the tissues was blocked by 30 min incubation with normal goat sera. Sections were then incubated with primary antibodies (1:200 dilution for ClC-7 antibody, 1:100 dilution for the anti-Ae2 antibody) or non-immune rabbit IgG (negative controls) overnight at 4 °C. After washing in TBS, sections were incubated with goat anti-rabbit-IgG-polymer conjugated with peroxidase (EnVision kit; Dako Cytomation, Glostrup, Denmark) for 1 h at ambient temperature (Henriksen et al. 2004; Schaller et al. 2004), washed and the peroxidase visualized using DAB (EnVision kit), counterstained with hematoxylin, dehydrated and mounted in Depex.

### Microcomputer tomography (micro-CT)

The heads and tibia from three *Clcn7*^-/-^ mice and three wild-type littermates were scanned under the same conditions at a resolution of 6 μm voxels using a μCT-40 high-resolution scanner (Scanco Medical, Bassersdorf, Switzerland). After scanning, 3-D computer reconstructions were made of the incisors, first molars, calvarial and jaw bones to detect structural changes in mineralized tissues in *Clcn7*^-/-^ mice. The 3-D reconstructions were done under the same threshold for wild-type and *Clcn7*^-/-^ mice. Cross-sectioned images of the incisors were collected at sequential intervals of 120 μm. In each slice, the mineral density of enamel was measured half-way into the enamel layer within a spot area with a diameter of 6 μm at the mesial, central and lateral side. The mineral density of the enamel, crown dentine, alveolar bone, calvarial and tibial bone was measured at three random sites per section and values averaged per slice. In the incisors, the values for enamel and dentin were plotted against slice number, which represented mineralization with increasing development (from secretory stage to maturation stage). Average values and standard deviation were calculated per mouse and these averages were used to calculate group averages. Since the incisors of the *Clcn7*^-/-^ mice did not erupt, for the incisors of wild-type controls, enamel values were used from the maturation stage of not yet erupted parts, to rule out post-eruptive changes when teeth are functional and exposed to oral fluids.

### Histomorphometric analysis

A point-counting technique (Cruz-Orive and Weibel 1990) was used to measure the bone volume (BV) and total tissue volume (bone volume + bone marrow volume, TV) of the maxillary bone (palatine bone) and mandibular bone (i.e., alveolar bone around molar region representing the alveolar region and condylar bone representing the ascending ramus, separately; Klingenberg et al. 2004).

### Statistical analysis

Values are presented as means and standard deviation (SD) and data were tested for statistical differences using Student’s *t* test and one-way ANOVA between the wild-type and *Clcn7*^-/-^ mice.

## Results

### Anti-ClC-7 immunostaining of ameloblasts and cells involved in bone remodeling

**Fig. 1:**
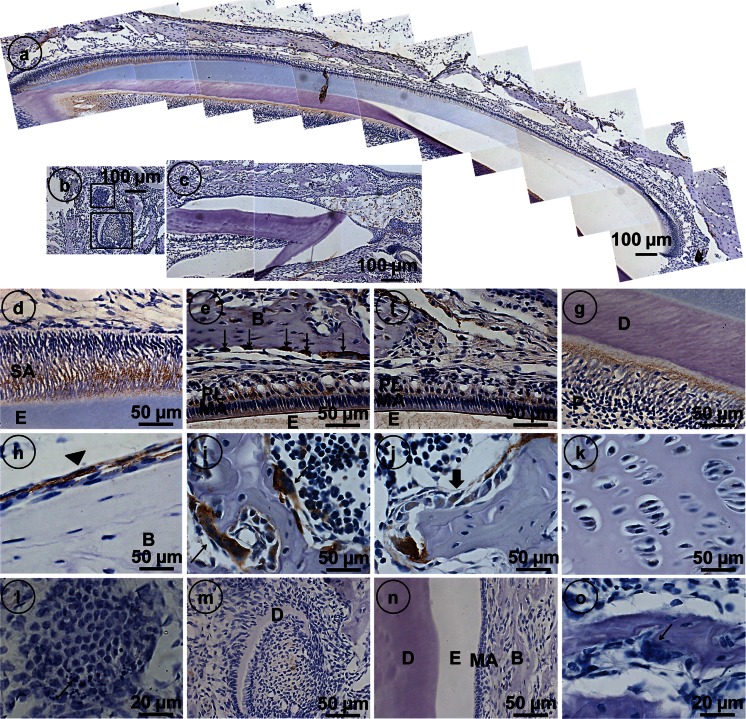
Immunolocalization of ClC-7 in developing jaw tissue of WT mice (**a**,** d**–**k**) and *Clcn*7^-/-^ mice (**b**,** c**,** l**–**o**). In WT mice, secretory ameloblast (*SA*) maturation ameloblasts (*MA*) and cells of the papillary layer (*PL*) stained positively in fine vesicular structures in the cytoplasm (**a**,** d**–**f**), whereas maturation ameloblasts also stained strongly in the apical part (*PL*) (**e**,** f**). Odontoblasts stained positively as well (**g**)* E* enamel,* D* dentin,* B* alveolar bone,* P* pulp;* Arrows* in (**e**) strongly stained osteoclasts. Bone lining cells indicated by *arrowheads* in (**h**) and osteoblasts indicated by* arrows* in (**j**) are all moderately stained in the cytoplasm. The chondrocytes are weakly stained in the cytoplasm (**k**). Intensely stained ruffled border of osteoclasts indicated by *arrows* are shown in (**i**). *Clcn*7^-/-^ mice are negative for ClC-7 immunostaining (**b**,** c**,** l**–**o**). ** b** The developing third molar of a *Clcn*7^-/-^ mouse. ** l**,** m** (magnified images of **b**) The abnormal apoptosis of enamel organ indicated by *arrows* in (**l**). **o** The negatively stained osteoclast in the *Clcn*7^-/-^ mice. Magnifications (**a**–**c**) ×200; (**d**–**k**,** m**,** n**) ×400; (**l**, **o**) ×1000.* Scale bars* (**a**–**c**) 100 μm, (**d**–**k**, **m**, **n**) 50 μm, (**l**, **o**) 20 μm

In jaws of wild-type mice, strong immunostaining with anti-ClC-7 was seen as fine granular material in secretory and maturation ameloblasts (Fig. 1a, d–f). Intense staining was noticed in osteoclasts (Fig. 1e, i) in the apical membrane of maturation ameloblasts and in papillary cells of the enamel organ. Intracellular staining was found in groups of maturation ameloblasts (Fig. 1e, f). Less intense staining was found in odontoblasts (Fig. 1g), bone lining cells (Fig. 1h), osteoblasts (Fig. 1j) and hypertrophic chondrocytes (Fig. 1k). Anti-ClC7 failed to stain enamel organ cells in incisors, developing third molars (Fig. 1b, c) or the giant osteoclasts bordering bone (Fig. 1o) in *Clcn7*^-/-^ mice. In developing third molars, the enamel organ contained cells with more pronounced apoptotic bodies than in wild-type controls (Fig. 1b, l).

### Immunolocalization of Ae2 in developing jaw tissues and long bones of ***Clcn***7^-/-^ mice

**Fig. 2:**
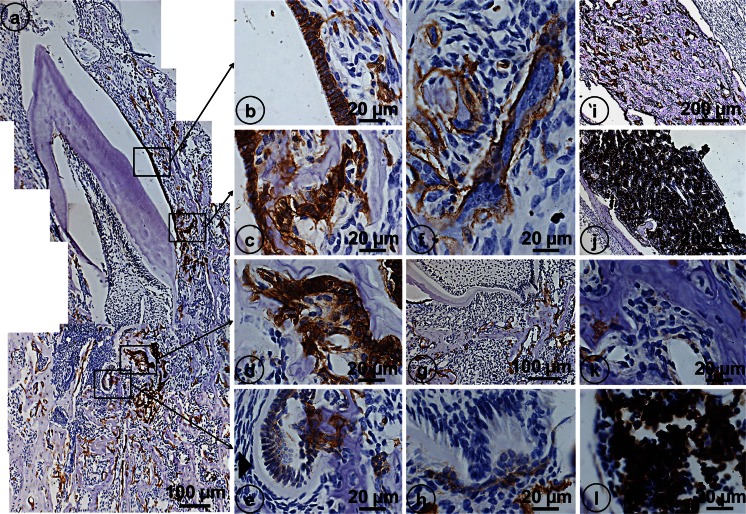
Immunolocalization of Ae2 in developing jaw tissues (**a**–**h**) and tibiae of *Clcn*7^-/-^ mice (**i**,** k**) and tibiae of wild-type mice (**j**,** l**). In *Clcn*7^-/-^ mice, maturation ameloblasts (*MA*) are strongly positively stained in the basolateral membrane (**b**,** c**). The odontoma‘s (*arrowhead*) trapped in the apical part of incisor are also positively stained (**c**–**e**). **f** A positively stained elongated osteoclast with many nuclei. The positively stained abnormal osteoclasts are elongated and attached along the surface of alveolar bone in the root of molars (**g**). The magnified images of (**g**) show the abnormal cervical loop of molars (**h**). In the large bone marrow cavity of WT mice but not in *Clcn*7^-/-^ mice, many cells are positive for Ae2.* Scale bars* (**a**, **g**) 100 μm, (**b**–**f**, **h**, **k**, **l**) 20 μm, (**i**, **j**) 200 μm

To see if disruption of ClC-7 also affected expression of anion exchanger 2 (Ae2), sections were immunostained for Ae2. The basolateral membranes of maturation ameloblasts of *Clcn7*^-/-^ mice were strongly positive for Ae2 (Fig. 2a–c) as reported in wild-type mice (Bronckers et al. 2009). In *Clcn7*^-/-^ mice, the layer of dental epithelium in the apical part of the incisors had been disrupted and ameloblasts and odontoblasts were trapped by alveolar bone forming odontoma-like structures but the dental epithelium strongly stained for Ae2 (Fig. 2c–e). Huge elongated multinucleated osteoclasts covering the bone surface near the distorted molar roots were also strongly stained for Ae2 (Fig. 2f, g). In both craniofacial bone and tibiae of wild-type mice, the extensive bone marrow cavities contained large numbers of mononuclear cells positive for Ae2. However, no such positive cells were seen in the population of marrow cells in the far less extensive marrow cavities either long bone (Fig. 2i, k) or craniofacial bones in *Clcn7*^-/-^ mice indicating the composition of the bone marrow cells had changed by disruption of *Clcn7* gene.

### Structural changes in craniofacial bones in ***Clcn***7^-/-^ mice

**Fig. 3:**
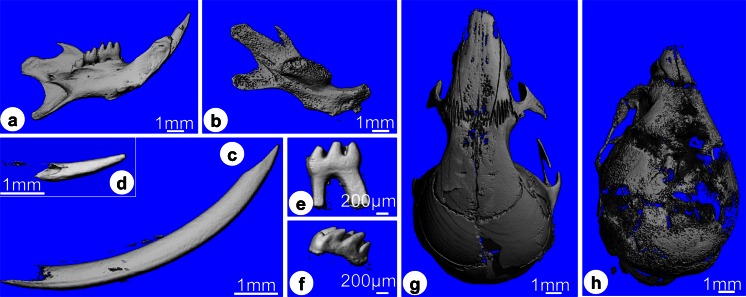
3-D reconstructed images by micro-CT of the mandibular jaw bones (**a**,** b**), mandibular incisors (**c**,** d**), first molars (**e**,** f**) and the calvarial bones (**g**,** h**). **a** (WT) and ** b** (KO) mandibular jaw bones are reconstructed under the same threshold. In the *Clcn*7^-/-^ mice, the incisors and molars are all impacted in the alveolar bone without eruption. The bone looks spongy and less mineralized. **c** (WT) and ** d** (KO) show the mandibular incisors. The wild-type incisor is much longer and better shaped than the mutant incisor with a blunt tip. The first molar crown of the mutant mice is well developed but no roots are formed (**f**). The calvarial bone has also the severe osteopetrotic phenotype such as jaw bones with a smaller dimension and lower mineral density than wild-type control mice (**g** WT;** h** KO).* Scale bars* (**a**–**d**, **g**, **h**) 1 mm, (**e**, **f**) 200 μm

**Fig. 4:**
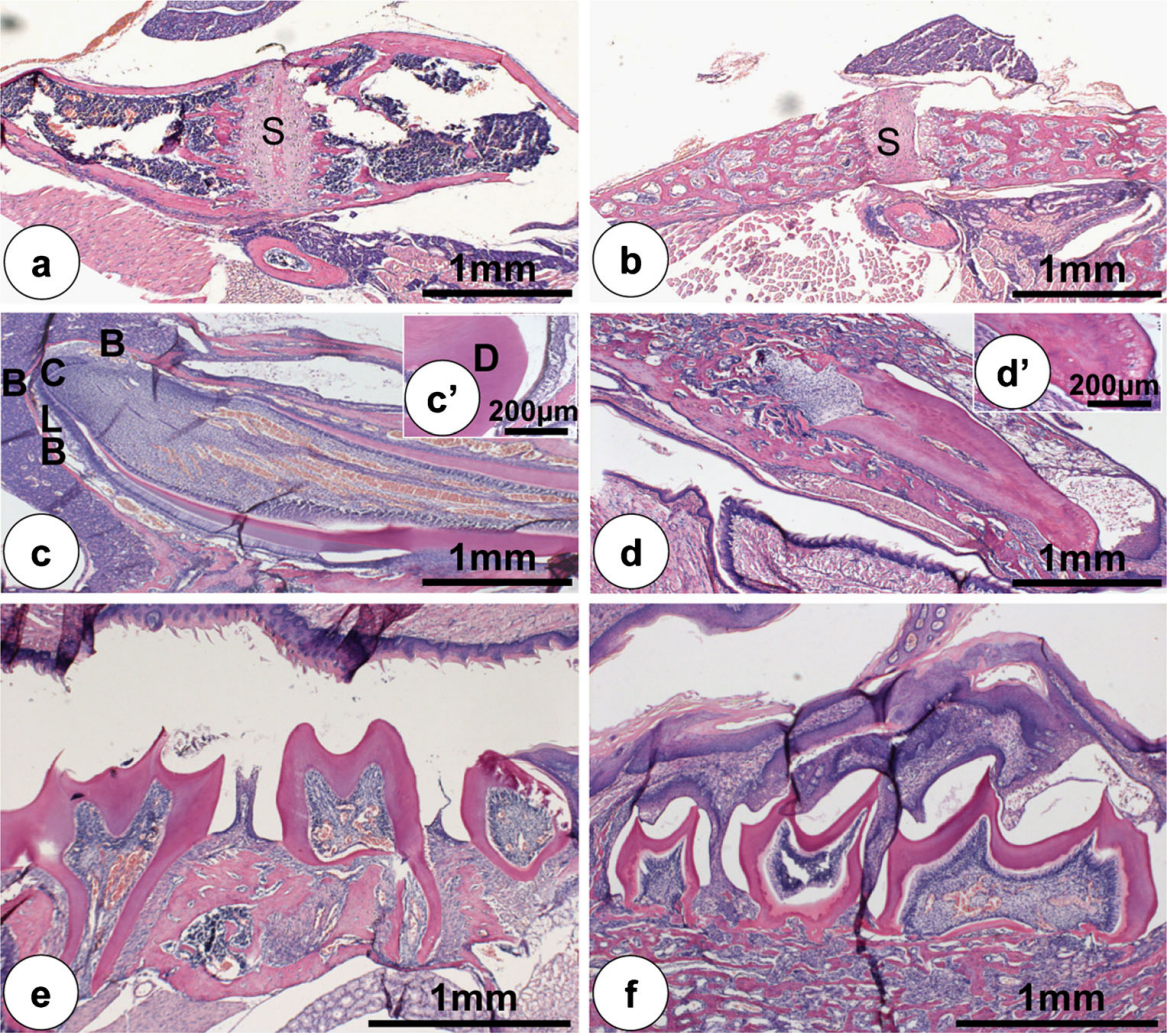
Histological changes of the jaw bones and teeth in *Clcn*7^-/-^ mice. Palatine bone with suture (*S*) (**a**,** b**). **a** In wild-type mice (WT), the bone is mature with large bone marrow cavities. **b** In *Clcn*7^-/-^ mice, the bone is osteopetrotic with many small marrow cavities. WT incisor with normal wide space between the cervical loop (*CL*) and bone (*B*) (**c**).The radiated arrangement of the normal dentinal tubules of wild-type incisor (**c’**). The *Clcn*7^-/-^ mice incisor with wide predentine and folded root (**d**) and the dentine (*D*) calcification is abnormal (**d’**). The molars of *Clcn*7^-/-^ mice failed to erupt with tissues covering the eruption route (**e** WT; **f** KO). Magnifications (**a**–**f**) ×50; (**c’**, **d’**) ×400. Hematoxylin staining. All magnifications indicate original magnification. *Scale bars* (**a**–**f**) 1 mm, (**c’**,** d’**) 200 μm

Micro-CT showed substantial differences in mineralized structures of jaw bones, skull and teeth between *Clcn7*^-/-^ and wild-type mice (Fig. 3). The bone surface of *Clcn7*^-/-^ mice appeared spongy compared to wild-type controls that had a denser and smoother surface (Fig. 3a, b, g, h). Histology showed that jaw bones in *Clcn7*^-/-^ mice exhibited a very severe osteopetrotic phenotype as reported for long bones (Fig. 4b) (Kornak et al. 2001). Jaw bones in *Clcn7*^-/-^ mice contained more trabeculae with many small marrow cavities instead of more mature bone with fewer but larger bone marrow cavities in wild-type controls (Fig. 4b). The trabecular structure of the palatine bone in *Clcn7*^-/-^ mice was disorganized. Osteocytes in bone of *Clcn7*^-/-^ mice were larger, round and arranged more irregularly than osteocytes in wild-type mice that were elongated with their long axes parallel to the bone surface. Many of the osteocyte lacunae in bones of *Clcn7*^-/-^ mice were empty suggesting enhanced apoptosis of osteocytes. The maxillary bone suture was closed in wild-type mice but not in *Clcn7*^-/-^ mice and retained substantial amounts of cartilage (Fig. 4a, b). The ratio of the width of zone of hypertrophic cartilage to the width of total suture area was 0.34 (±0.03) for wild-type mice and 0.62 (±0.04) for *Clcn7*^-/-^ mice (*n* = 3, *p* < 0.001).

**Fig. 5:**
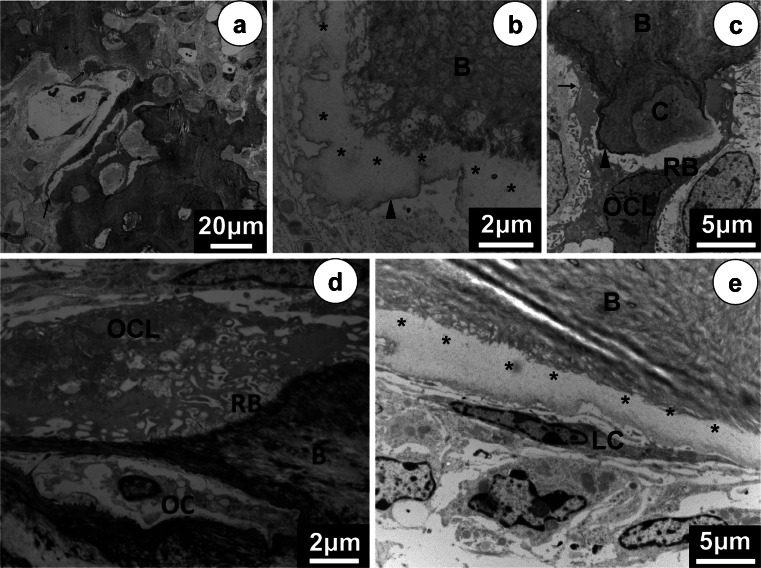
Ultrastructural changes in osteoclasts and in the structure of craniofacial bones in *Clcn*7^-/-^ mice. **a** A huge elongated osteoclast covers a large area of the bone forming many sealing zones on the bone surface in the *Clcn*7^-/-^ mice.* Arrows* (**a**,** c**) indicate the extensive sealing zone of the osteoclast. **b** The poorly matured jaw bone (*B*) in the *Clcn*7^-/-^ mice with cementum-like-line structure (*arrowhead* in **b**,** c**) and amorphous layer (*asterisk*) between cell and fibrillar bone (*B*) consisting of loosely packed collagen bundles. Comparing the osteoclasts of the *Clcn*7^-/-^ mice (**c**) and that of the WT mice (**d**), the ruffled border (*RB*) of the osteoclast (*OCL*) from *Clcn*7^-/-^ mice is almost not developed. **e** The abnormal amorphous material between lining cells (*LC*) and calcified bone (*B*) in the *Clcn*7^-/-^ mice.* OC* osteocyte,* C *calcified cartilage.* Scale bars* (**a**) 20 μm, (**b**, **d**) 2 μm, (**c**, **e**) 5 μm

Electron microscopy revealed extremely long osteoclasts in *Clcn7*^-/-^ mice containing broad clear zones, many intracellular vesicles and a ruffled border that was poorly developed (Fig. 5a, c). Undecalcified samples of craniofacial bone of the *Clcn7*^-/-^ mice were easier to section. Collagen bundles of the bone matrix were very loosely packed and between osteoblasts and collagen fibrils of the bone matrix substantial amounts of amorphous non-fibrous material was present (Fig. 5b, e), not seen in wild-type bone (Fig. 5d). The bone surface contained more frequently prominent cementum-like lines to which elongated osteoclasts were attached (Fig. 5b, c). Osteoclasts seemed to resorb some bone and calcified cartilage but far less than controls (Fig. 5a, c).

Bone volume of the *Clcn7*^-/-^ mice was 1.53 fold higher in palatine bone (*p* < 0.001), 1.61-fold higher in alveolar bone (0.01 < *p* < 0.05) and 1.43-fold higher in condyle (0.01 < *p* < 0.05) than the corresponding parts in wild-type control mice (Table 1).

**Table 1:** Histomorphometric measurement of the ratio of bone volume and total volume in different craniofacial bone regions (MV±SD)

Location	Wild-type mice	*Clc*-7^-/-^ mice	Ratio of KO/WT
Palatine bone	36.6 ± 3.3 %	55.9 ± 2.9 %	1.53***
Alveolar bone	38.1 ± 5.6 %	61.2 ± 9.8 %	1.61*
Condyle	37.2 ± 2.9 %	53.2 ± 9.5 %	1.43*

### Structural changes in developing teeth

In contrast to wild-type controls, incisors and molars of *Clcn7*^-/-^ mice failed to erupt into the oral cavity (Figs. 3b, 4d). Incisors were often still surrounded by dense bone, incisors were shortened by two-third in comparison with wild-type controls (Fig. 3c, d), but the dental crowns of the molars developed well. However, root formation of the molars was distorted and disfigured by compaction (Fig. 3e, f). In decalcified teeth, no enamel matrix was left in the enamel space and the space was bordered by flattened ameloblasts. Dentine mineralization was irregular, predentine was wider and the dentine tubules were narrower. Parts of incisor dentine were hypomineralized and contained irregular dentin tubules. At the cervical loop of the *Clcn7*^-/-^ incisors, the dental epithelium layer with the proliferating cells had folded, disrupted and fragments of the layer containing ameloblasts and odontoblasts formed many islands surrounded by bone tissue. Ankylosis occurred locally between dental roots and alveolar bone. Periodontal ligament was poorly developed and the enamel organ occasionally formed cysts (Fig. 4c, d).

### Changes in mineral density

**Fig. 6:**
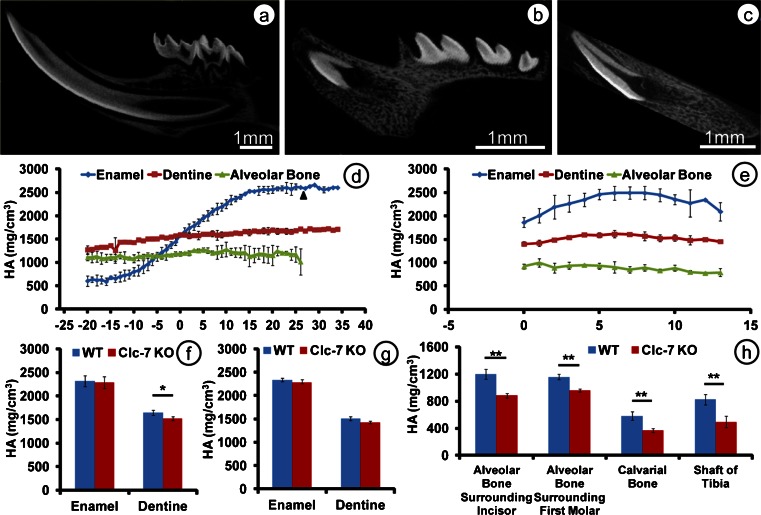
Mandibular jaw bones of wild-type mice (**a**) and *Clcn*7^-/-^ mice (**b**,** c**) in a sagittal plane from a 3-D reconstructed micro-CT image. Effect of *Clcn*7 null mutation on mineral density of developing lower incisors as function of stage (**d** wild-type mice; **e** *Clcn*7^-/-^ mice).* 0* on* x-axis* represents the starting point of maturation stage. Average mineral density of enamel and dentine in maturation stage exclusive the erupted part for wild-type incisors but the whole incisor of *Clcn*7^-/-^ mice (**f**) and first molars (**g**). Average mineral density in alveolar and calvarial bones (**h**). Scale bars (**a**–**c**) 1 mm

In the *Clcn7*^-/-^ mice, the calcified part of the mandibular incisor was much shorter but the mineral density of enamel still reached values as high as 2493 mg HA per cm^3^, not statistically different from control values that reached maximal values of 2630 mg HA/cm^3^ (Fig. 6d–g). Mineral density of incisor dentine was slightly less than the controls (*p* < 0.05).

The mineral density of the bone in *Clcn7*^-/-^ mice was less: in calvaria: 36.4 % lower than in wild-type mice (0.001 < *p* < 0.01), in alveolar bone in the incisor region: 26.2 % lower (0.001 < *p* < 0.01) and near the first molar region: 17.2 % lower (0.001 < *p* < 0.01) (Fig. 6h).

## Discussion

Our data show that ClC-*7* is critical for osteoclast functioning in orofacial and calvarial bone as reported for long bones (Kornak et al. 2001; Neutzsky-Wulff et al. 2008). Without functional ClC-*7*, orofacial bones became severely osteopetrotic and teeth failed to erupt. Impaction of the teeth, root dysplasia and odontoma-like structures were regularly noticed in various osteopetrotic models. So far, these characteristics have been found in five osteopetrotic models, namely *ntl*^-/-^, *Rank*^-/-^ , *Rankl*^-/-^, *c*-*src*^-/-^ and *v*-*H*^+^-*ATPase*^-/-^ mice (Bronckers et al. 2012; Koehne et al. 2013; Lu et al. 2009; Tiffee et al. 1999). Two *Clcn7*-related osteopetrotic patients suffered from enamel dysplasia and root dysplasia in posterior teeth (Xue et al. 2012). Osteopetrotic cattle with a *Clcn7* mutation displayed gross gingival hamartomas, not found in *Clcn7*^-/-^ mice (Sartelet et al. 2014). We reported here a strong positive immunostaining of wild-type ameloblasts for ClC-7 in line with published data (Lacruz et al. 2013). We also found changes of the dentition (including enamel, dentin, roots and periodontal tissues) in *Clcn7*^-/-^ mice. We found no evidence that without functional ClC-7 mineralization of enamel was reduced or enamel matrix retained as seen in *Cftr*^-/-^ (Wright et al. 1996) or *Ae2*^-/-^ mice (Lyaruu et al. 2008). Basically, the same structural changes in teeth were reported very recently in *Clcn7*^-/-^ mice in a less extensive way while we prepared this manuscript (Wen et al. 2015). Disruption of *Ae2* affects both osteoclasts in long bone and maturation ameloblasts (Jansen et al. 2009). Ameloblasts in *Clcn7*^-/-^ mice stained normally for Ae2 and were not structurally affected. Normal immunostaining for Ae2 was also noticed in basolateral membranes of defective osteoclasts in *Clcn7*^-/-^ mice, suggesting that osteoclasts expression of Ae2 was not affected and that these osteoclasts are potentially able to import Cl^−^ from the extracellular fluid.

Although teeth failed to erupt, the enamel of *Clcn7*^-/-^ mice attained the same mineral density as in wild-type enamel and enamel matrix was not retained. These data indicate that ClC-7 is not significantly involved in completion of enamel mineralization as found for ameloblasts deficient for MMP20, KLK4, SPPL2a CFTR, AE2 or NBCe1 (Bartlett et al. 2004; Bronckers et al. 2010, 2013; Jalali et al. 2014; Lyaruu et al. 2008; Simmer et al. 2009).

In osteoclasts, ClC-7 operates in conjunction with the proton pump associated with the ruffled border, for which the subunit Tcirg1 (T-cell immune regulator 1, also called Atp6v_0_a_3_) is essential. Both *Clcn7*^-/-^ mice and v-H^+^-ATPase null mutation gave an osteopetrotic phenotype but without marked changes in ameloblast function or in enamel mineralization (Bronckers et al. 2012; Wen et al. 2015). Thus, unlike their function in osteoclasts, ClC-7 or v-H^+^-ATPase are not critical for ameloblast function.

Osteopetrosis is a heterogeneous group of genetic bone disease characterized by an increase in bone volume due to the absence or an impaired activity of osteoclasts (Tolar et al. 2004). Changes in bone formation and in degree of mineralization of osteopetrotic bone, which also influences bone quality (Bollerslev 1989; Tolar et al. 2004; Waguespack et al. 2007), received less attention. In wild-type mice during endochondral ossification, the cartilage is gradually replaced by bone and old bone replaced by new bone (Seeman and Delmas 2006). For *Clcn7*^-/-^ mice, we found that bone resorption and bone-turnover were reduced and cartilage was retained. The amount of bone was higher in *Clcn7*^-/-^ mice but the bone was less mineralized and collagen fibrils less packed and were covered by a layer of amorphous non-collagenous material. This suggested that in vivo mineralization of bone was also impaired. However, in vitro data showed that the ability of osteoblasts of *Clcn7*^-/-^ mice to mineralize was *not* altered (Henriksen et al. 2011), suggesting that the changes in bone structure in vivo may be secondary or that in vivo osteoblasts respond differently.

The lower mineral density of bone in *Clcn7*^-/-^ mice implies that the bone quality and hence the capacity to carry mechanical loads may be reduced in these mice (Del Fattore et al. 2008; Henriksen et al. 2011; Segovia-Silvestre et al. 2009). In young postnatal *Clcn7*^-/-^ mice, the bone mineral density was lower and the bone contained empty osteocyte lacunae. However, in adult *Clcn7*^-/-^ mice, bone strength was increased as compared to bone in wild-type mice (Henriksen et al. 2011), suggesting that the changes in bone in *early postnatal Clcn7*^-/-^ mice could be transient. Apparently, the increased amount of bone mass in *Clcn7*^-/-^ mice outweighs the reduction in mineral content per unit of bone. However, patients with defective ClC-7 appear to have poor bone quality and suffer from many fractures despite their higher bone mass. This may be related to elevated amounts of calcified cartilage present in bone that weakens the bone structure.

The most commonly affected genes of osteopetrosis are CLCN7 and TCIRG1. Osteopetrosis is frequently accompanied by osteomalacia or osteopetrorickets (Barvencik et al. 2014) and this additional pathology is also found in 2-week-old *Tcirg1*-deficient *oc*/*oc* mice. This phenotype was characterized by growth retardation, rachitic widening of the growth plate, marked hyperosteoidosis and diffuse mineralization of the bone surfaces. These defects are the consequence of hypocalcemia resulting from a combined acidification, impairment of osteoclasts and gastric parietal cell. *Clcn7*^-/-^ mice have some of these characteristics such as growth retardation and widening of the hypertrophic zone in the growth plate. However, hyperosteoidosis was *not* detected in *Clcn7*^-/-^ mice nor did these mice display hypocalcemia. Although our micro-CT data showed that the bone mineral density in *Clcn7*^-/-^ mice was significantly lower than the bones in the wild-type mice, *Clcn7*^-/-^ mice do not display clear osteopetrorickets at the age of 3 weeks. A novel mutation in CLCN7 (D145 G) impaired the activation and relaxation kinetics of the CLC-7 ion transporter and had no sign of osteomalacia (Barvencik et al. 2014). Thus, in contrast to patients carrying TCIRG1 mutation, patients carrying CLCN7 mutation do not seem to suffer from osteomalacia.

In conclusion, ClC-7 is essential for osteoclasts to resorb craniofacial bones to enable tooth eruption and root development. Disruption of *Clcn7* reduces bone and dentin mineral density but does not affect enamel mineralization.
